# Machine Learning Approach to find the relation between Endometriosis, benign breast disease, cystitis and non-toxic goiter

**DOI:** 10.1038/s41598-019-41973-w

**Published:** 2019-04-01

**Authors:** Jung Hun Lee, Seon-Young Kwon, Jiho Chang, Jin-Sung Yuk

**Affiliations:** 1 Department of Obstetrics and Gynecology, Ewha Womans University Seoul Hospital, School of Medicine, Ewha Womans University, Seoul, Republic of Korea; 2 Department of Family Medicine, Yonsei Spring Clinic, Namyangju-si, Republic of Korea; 3 Coupang Korean Electronic Commerce Company, Seoul, Republic of Korea; 4 Department of Obstetrics and Gynecology, Eulji University College of Medicine, Nowon Eulji Medical Center, Seoul, Republic of Korea

## Abstract

The exact mechanism of endometriosis is unknown. The recommendation system (RS) based on item similarities of machine learning has never been applied to the relationship between diseases. The study aim was to identify diseases associated with endometriosis by applying RS based on item similarities to insurance data in South Korea. Women aged 15 to 45 years extracted from the Korean Health Insurance Review & Assessment Service National Inpatient Sample (HIRA-NIS) 2009–2015. We used the RS model to extract diseases that were correlated with an endometriosis diagnosis. Among women aged 15 to 45 years, endometriosis was defined as a diagnostic code of N80.x and a concurrent treatment code. A control group was defined as women who did not have the N80.x code. Benign breast diseases, cystitis, and non-toxic goitre were extracted by the RS. A total of 1,730,562 women were selected as the control group, and 11,273 women were selected as the endometriosis group. In logistic regression analysis adjusted for age per 5 years, data year, and socioeconomic status, benign neoplasm of breast (odds ratio (OR): 2.58; 95% confidence interval (CI): 1.90–3.50), other cystitis (OR: 2.63; 95% CI: 1.56–4.44), and non-toxic single thyroid nodule (OR: 1.62; 95% CI: 1.14–2.32) were statistically significant. Endometriosis was associated with benign breast disease, cystitis, and non-toxic goitre.

## Introduction

Endometriosis is a disease in which the endometrial gland and stromal tissue in the endometrium are located outside the uterine cavity. Additionally, endometriosis is an estrogen-dependent disease that causes symptoms of dysmenorrhoea, infertility, and abnormal uterine bleeding^1^.

Retrograde transplantation theory is a commonly accepted hypothesis for the pathogenesis of endometriosis. This theory suggests that endometrial cells flow into the peritoneal cavity through the fallopian tube during menstruation and attach to the peritoneal surface^1^. Most women have menstrual regurgitation, but only 6–10% of women have endometriosis^1^. In fact, the exact mechanism of endometriosis is unknown. Several studies have shown that some autoimmune diseases are related to endometriosis^2–4^. These studies have provided insight to the immunological alterations that may influence the development of endometriosis. Additionally, according to a meta-analysis, endometriosis is strong risk factor for ovarian cancer^5,6^. These findings have led to several hypotheses about the development of ovarian cancer, in which genetic or non-genetic factors may transform endometriotic endometrial tissues to malignant tissue^7^. An examination of the relationships between endometriosis and other diseases could help clarify the pathogenesis of endometriosis and other diseases. However, selecting a candidate disease to study in association with endometriosis will be entirely dependent on previous research or individual experience.

**Figure 1:**
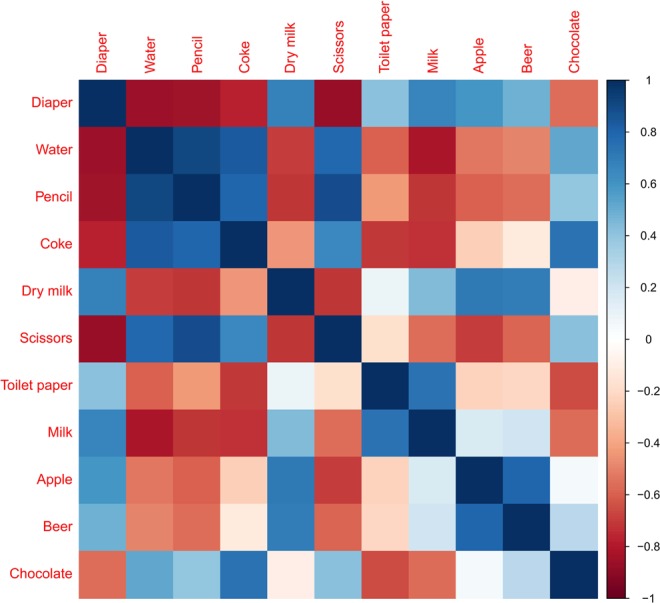
Similarity matrix between items. Background colours of cells indicate the similarity between the two items. A stronger blue colour indicates a higher similarity between the two items.

A recommendation system (RS) is a type of machine learning system that recommends what a person may want to purchase on Amazon or watch on Netflix^8^. For example, suppose a new user purchased diapers on Amazon. The goal of Amazon is to know what other products a new user might be interested in to increase sales. To do this, Amazon analyzes the purchases of other users. Amazon knows that other users who have purchased diapers are more likely to buy milk powder and thus can recommend milk powder to the new user. This recommendation can be expressed as a similarity matrix with items in rows and columns (Fig. 1), and it is called RS based on item similarities^8^. The RS for items sold in the store had a matrix structure. Similarly, the correlation of diagnostic codes applied to patients also has a matrix structure. Therefore, endometriosis can be substituted for diapers, and RS can be used to estimate diseases associated with endometriosis.

The purpose of this study is to identify diseases associated with endometriosis by applying RS based on item similarities to claim sample data in South Korea.

## Material and Methods

### Samples

National Health Insurance Service (NHIS) in South Korea covers approximately 98% of all Koreans living in South Korea. The NHIS supports the medical costs of most diseases, with a few exceptions, such as cosmetic procedures^9^. Health Insurance Review & Assessment Service (HIRA) is an agency that evaluates medical expenses charged by medical institutions, and it shares most related insurance information to the NHIS^9^. The HIRA provided sample data, called the Health Insurance Review & Assessment Service-National Inpatient Sample (HIRA-NIS), to researchers, which included various insurance information, such as sex, age, prescription history, medical test history, surgical history, and diagnostic code {the International Statistical Classification of Diseases and Related Health Problems 10^th^ edition (ICD-10)}^9^. HIRA-NIS extracted data for 1 million people per year from approximately 49 million subjects using a probabilistic weighted sample extraction method (13% of patients admitted once to a medical institution in a year and 1% of patients who had never been hospitalized)^9^. HIRA-NIS showed no difference in the disease incidence compared to the total claim data of the HIRA^10^. HIRA-NIS data can be requested in the HIRA data site (http://opendata.hira.or.kr). The data in our study did not has any other data linkage with the exception of HIRA-NIS. Medical care patients who received government support were classified into a low socioeconomic status (SES) group.

### Selection of candidate diseases associated with endometriosis

Patients admitted to the medical institution once a year were extracted from the HIRA-NIS 2009–2015 (Serial keys: 2009-0066//2010-0084//2011-0063//2012-0058//2013-0085//2014-0068//2015-0057). Of the total data, 80% was randomly extracted to create an RS model. The remaining 20% was verified for this model. The RS model was constructed using the first three-character categories of the ICD-10 of these patients {e.g.; N80 from N80.1 (Endometriosis of ovary), N30 from N30.0 (Acute cystitis)}. The RS model was used to select 30 endometriosis related diseases. The reasons for the limitation of 30 diseases were arbitrarily determined considering the efficiency of the study.

**Figure 2:**
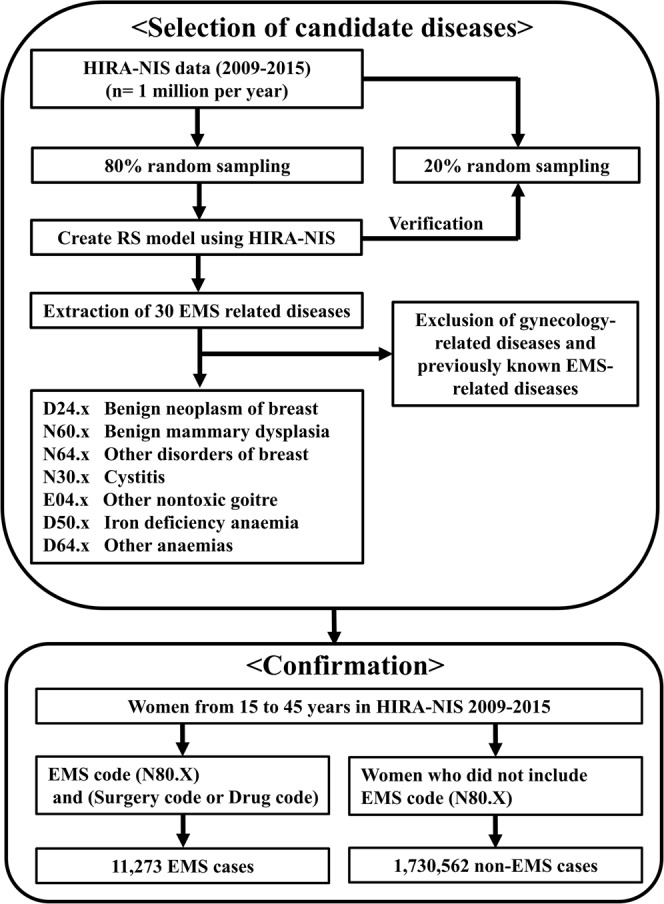
Flowchart creating a recommender model using HIRA-NIS data. HIRA-NIS: Health Insurance Review & Assessment Service National Inpatient Sample.

Patients with endometriosis underwent physical examination and ultrasonography by a gynaecologist. These gynecologic examinations may detect asymptomatic gynecologic diseases such as leiomyoma, ovarian cysts or vaginitis, which can generate bias. Thus, gynecologic diseases [C51~C58 (Malignant neoplasm of female genital organs), D06~D07 (Carcinoma *in situ* of cervix uteri, other and unspecified genital organs), D25~D28 (Benign neoplasm of uterus, ovary, other and unspecified female genital organs), O00~O99 (Pregnancy, childbirth and puerperium), N70~N77 (Inflammatory diseases of female pelvic organs), N80~N98 (Noninflammatory disorders of female genital tract)] were excluded from the 30 endometriosis-related diseases (Fig. 2).

### Confirmation of the relationship between endometriosis and candidate diseases

Data from women aged 15 to 45 years were extracted from the HIRA-NIS 2009–2015. Among these women, those with endometriosis were defined as having a diagnostic code of N80.x and concurrent gynecologic surgery or concurrent prescription (Fig. 2).

The control group was defined as 15- to 45-year-old women who did not have the diagnostic code N80.x in the HIRA-NIS 2009–2015. A total of 1,730,562 women were selected as the control group, and 11,273 women were selected as the endometriosis group.

Candidate diseases selected through the RS model in the endometriosis and control groups were defined by a combination of the diagnostic code and clinically valid test code in the candidate disease.

The generated data used in our study is provided in supplementary information files.

### Statistics

Python version 2.7.13 (Python Software Foundation, Oregon, USA) was used for RS of machine learning. RS based on item similarities using the Jaccard index, which is a preferred similarity index for data without human estimations or ratings, was used to calculate similarities between diseases. R version 3.3.2 (The R Foundation for Statistical Computing, Vienna, Austria) was used for all statistical calculations. A chi-square test and Fisher’s exact test were used to compare categorical variables (data year and presence or absence of each disease), and a weighted t-test was performed for mean comparison of continuous variable (age). Weighted logistic regression was used to adjust categorical variables by age per 5 years and sample year. We used weighted parameters (inpatient group: 7.692; outpatient group: 100) in the HIRA-NIS for weighted analysis. Statistical significance was defined as having a p-value less than 0.05, and all statistical hypothesis tests were performed using a two-sided test. For missing values, the mean imputation method was used.

### Ethical statement

This cross-sectional study received Institutional Review Board (IRB) approval at Gyeongsang National University Changwon Hospital (IRB No. 2017–03–002) (the date of approval: April 20, 2017). This study does not disadvantage the involved individuals because the data did not contain personally identifiable information. Therefore, this study did not require informed consents from subjects under the South Korea’s Bioethics and Safety Act. This study was conducted in accordance with the guidelines of the South Korea’s Bioethics and Safety Act.

## Results

### Selection of candidate diseases associated with endometriosis

The RS model was created using 80% (random sampling) of HIRA-NIS data from 2009 to 2015. The RS model was validated for the remaining 20% of the HIRA-NIS; the mean precision was 0.032, and the mean recall was 0.036. In this model, 30 endometriosis-related disease categories were extracted by calculating the similarity to endometriosis (N80.x) (Supplement Table 1). Among these 30 disease categories, 7 disease categories were selected after excluding gynaecology-related diseases (Supplement Table 2) (Fig. 2).

### Confirmation of the relationship between endometriosis and candidate diseases

Mean ages were 30.8 ± 0.0 years in the control group and 34.1 ± 0.1 years in the endometriosis group, respectively (Table 1). The rate of endometriosis between ages15 and 44 years was 1.5 per 1,000. Women with benign breast disease were defined as simultaneously having a diagnostic code for benign breast disease [D24.x (Benign neoplasm of breast), N60.x (Benign mammary dysplasia), and N64.x (Other disorder of breast)] and breast examination codes (mammography, digital breast tomosynthesis, and breast ultrasound) (Table 1). Women with cystitis were defined as those who simultaneously had a cystitis diagnostic code [N30.x (Cystitis)] and urine test codes (urinalysis, microscopic examination for microorganism, microorganism primary culture test) (Table 1). Women with non-toxic goitre were defined as simultaneously having a non-toxic goitre diagnostic code [E04.x (Other non-toxic goitre)] and a thyroid test code (thyroid stimulating hormone (TSH), free T3, free T4, anti-thyroid antibody, anti-thyroglobulin antibody, and neck ultrasound) (Table 1). Women with iron deficiency anaemia or other anaemias were defined as having an anaemia diagnostic code [D50.x (Iron deficiency anaemia) or D64.x (Other anaemias)] and haemoglobin test codes (Table 1).

**Table 1:** Characteristics of endometriosis and control groups.

	Control	Endometriosis	P-value
Number of patients	1,730,562	11,273	
Mean age, year	30.8 ± 0.0	34.1 ± 0.1	<0.01^a^
Low SES	47,873 (2.8%)	144 (1.3%)	<0.01
Data year			0.01
2009	249,171 (14.4%)	1,564 (13.9%)	
2010	249,849 (14.4%)	1,684 (14.9%)	
2011	250,509 (14.5%)	1,622 (14.4%)	
2012	247,925 (14.3%)	1,682 (14.9%)	
2013	240,694 (13.9%)	1,460 (13.0%)	
2014	245,698 (14.2%)	1,653 (14.7%)	
2015	246,716 (14.3%)	1,604 (14.4%)	
Benign neoplasm of the breast	10,368 (0.6%)	137 (1.2%)	<0.01
Benign mammary dysplasia	6,255 (0.4%)	82 (0.7%)	<0.01
Other disorders of the breast	10798 (0.6%)	138 (1.2%)	<0.01
Cystitis	80,222 (4.6%)	742 (6.6%)	<0.01
Other non-toxic goitre	13,702 (0.8%)	119 (1.1%)	<0.01
Iron deficiency anaemia	20230 (1.2%)	328 (2.9%)	<0.01
Other anaemias	8947 (0.5%)	105 (0.9%)	<0.01

SES, socioeconomic status.

Diseases with a prevalence of less than 0.1% in both groups are not shown in the table.

^a^A weighted t-test was used.

In the logistic regression analysis using the first three-character categories of the ICD-10, benign breast diseases, cystitis, non-toxic goitre, iron deficiency anaemia, and other anaemias were significantly correlated with endometriosis (Table 2) (Supplement Table 3). Candidate diseases were divided into full diagnostic codes, and logistic regression analyses for each disease were performed after adjusting for age per 5 years, data year, and SES. Benign neoplasm of the breast, diffuse cystic mastopathy, unspecified benign mammary dysplasia, other signs and symptoms in the breast, unspecified disorder of the breast, acute cystitis, other cystitis, unspecified cystitis, non-toxic single thyroid nodule, non-toxic multinodular goitre, iron deficiency anaemia secondary to chronic blood loss, other iron deficiency anaemias, iron deficiency anaemia, unspecified, other specified anaemias, and unspecified anaemia were significantly correlated with endometriosis (Supplement Table 4). Logistic regression analysis was performed after adjusting for age per 5 years, data year, and SES with only statistically significant diseases in the previous analysis, and Table 3 shows the detailed results.

**Table 2:** Logistic regression analysis of endometriosis-related candidate diseases using middle-class diagnostic codes.

	Unadjusted^a^		Adjusted Model^b^	
	OR (95% CI)	P-value	OR (95% CI)	P-value
Age per 5 years			1.26 (1.24–1.28)	<0.01
Data year			1.01 (0.99–1.02)	0.38
Low SES			0.58 (0.44–0.77)	<0.01
Benign neoplasm of the breast	3.48 (2.57–4.72)	<0.01	2.58 (1.90–3.51)	<0.01
Benign mammary dysplasia	2.68 (1.83–3.92)	<0.01	1.92 (1.31–2.82)	<0.01
Other disorders of the breast	2.23 (1.71–2.92)	<0.01	1.76 (1.35–2.30)	<0.01
Cystitis	1.66 (1.46–1.88)	<0.01	1.51 (1.33–1.71)	<0.01
Other non-toxic goitre	1.95 (1.45–2.62)	<0.01	1.54 (1.15–2.08)	<0.01
Iron deficiency anaemia	3.48 (2.87–4.22)	<0.01	3.05 (2.51–3.72)	<0.01
Other anaemias	2.52 (1.82–3.47)	<0.01	2.08 (1.49–2.89)	<0.01

CI, confidence interval; OR, odds ratio; SES, socioeconomic status.

^a^ORs were analysed for endometriosis and each disease without other adjustments.

^b^Analysis was adjusted for all variables in the table (endometriosis ~ age per 5 years + data year + low SES + benign neoplasm of breast + benign mammary dysplasia + other disorders of the breast + cystitis + other non-toxic goitre + iron deficiency anaemia + other anaemias).

**Table 3:** Logistic regression analysis of endometriosis-related candidate diseases using full diagnostic codes.

	Unadjusted^a^		Adjusted Model^b^	
	OR (95% CI)	P	OR (95% CI)	P
Age per 5 years			1.26 (1.24–1.28)	<0.01
Data year			1.01 (0.99–1.02)	0.38
Low SES			0.58 (0.44–0.77)	<0.01
Benign neoplasm of the breast	3.48 (2.57–4.72)	<0.01	2.58 (1.90–3.50)	<0.01
Diffuse cystic mastopathy	3.17 (1.45–6.89)	<0.01	2.24 (1.03–4.88)	0.04
Benign mammary dysplasia	3.76 (1.92–7.4)	<0.01	2.66 (1.35–5.24)	<0.01
Other symptoms in the breast	3.62 (2.01–6.52)	<0.01	2.91 (1.61–5.24)	<0.01
Other disorders of the breast	2.65 (1.45–4.85)	<0.01	1.96 (1.07–3.60)	0.03
Unspecified disorder of the breast	2.83 (1.98–4.03)	<0.01	2.03 (1.42–2.91)	<0.01
Acute cystitis	1.51 (1.31–1.74)	<0.01	1.34 (1.16–1.54)	<0.01
Other cystitis	3.13 (1.87–5.26)	<0.01	2.63 (1.56–4.44)	<0.01
Unspecified cystitis	2.02 (1.55–2.64)	<0.01	1.70 (1.31–2.22)	<0.01
Non-toxic single thyroid nodule	2.01 (1.47–2.99)	<0.01	1.62 (1.14–2.32)	<0.01
Non-toxic multinodular goitre	2.15 (1.36–3.4)	<0.01	1.60 (1.01–2.53)	0.05
IDA secondary to blood loss	6.87 (3.88–12.15)	<0.01	5.30 (2.96–9.50)	<0.01
Other IDA	2.96 (1.96–4.46)	<0.01	2.31 (1.52–3.52)	<0.01
Unspecified IDA	3.37 (2.68–4.23)	<0.01	2.82 (2.23–3.56)	<0.01
Other specified anaemias	3.16 (1.88–5.31)	<0.01	2.50 (1.48–4.24)	<0.01
Unspecified anaemia	2.48 (1.74–3.53)	<0.01	2.03 (1.41–2.93)	<0.01

CI, confidence interval; OR, odds ratio; SES, socioeconomic status; IDA, iron deficiency anaemias.

^a^ORs were analysed for endometriosis and each disease without other adjustments.

^b^Analysis was adjusted for all variables in the table (endometriosis ~ age per 5 years + data year + low SES + benign neoplasm of breast + diffuse cystic mastopathy + unspecified benign mammary dysplasia + other signs and symptoms in the breast + other specified disorders of the breast + unspecified disorder of the breast + acute cystitis + other cystitis + unspecified cystitis + non-toxic single thyroid nodule + non-toxic multinodular goitre + iron deficiency anaemia secondary to blood loss + other iron deficiency anaemias + unspecified iron deficiency anaemia + other specified anaemias + unspecified anaemia).

## Discussion

### Main Findings

This study suggests that endometriosis may be associated with benign breast disease, cystitis, and non-toxic goitre through the use of RS in machine learning. In the confirmation analysis of the relationship between endometriosis and diseases recommended by the RS, benign breast disease, cystitis, and non-toxic goitre were associated with endometriosis.

RS based on item similarity is a research method that has not previously been used to confirm the relationship between diseases. RS suggested that seven three-character categories of the ICD-10 were associated with endometriosis. After confirmation, these categories were all associated with endometriosis in claim data. RS for endometriosis worked well in this study.

The first three-character categories of the ICD-10 were used for RS in this study. Several diseases have been associated with endometriosis when full diagnostic codes were applied, which are more subdivided than the first three-character categories diagnostic code. Additionally, although RS was useful in predicting the relationships between diseases, it was applied using the diagnostic code alone without a drug code or surgical code. This suggested that RS based on item similarity may be useful for confirming the relations between diseases using only the first three-character categories diagnostic code. Therefore, if RS is applied to non-gynecologic diseases, it could help identify the relationship between diseases and clues of pathogenesis. Further studies about other diseases are needed.

Previous studies questioned the relationship between specific diseases and endometriosis based on individual experience or from previous studies^2–4,11^. However, such a method has several disadvantages. First, the number of patients one physician can evaluate in a lifetime is limited. Second, it is difficult to find any association with endometriosis in diseases with low incidence or with different ages of onset. Third, if the related diseases are treated by different medical specialties (e.g., internal medicine, general surgery, and gynaecology), it is difficult for one medical specialist to question the relevance of the diseases.

Machine learning is a useful way to overcome these drawbacks. Machine learning is a part of artificial intelligence (AI), a scientific discipline in which computers automatically learn using given data^12^. It is used in many fields, such as spam filtering, computer vision to read MRIs, bioinformatics, speech recognition, autonomous driving, translation, etc., because it is useful for quickly and easily evaluating massive data that are difficult for humans to analyze^12,13^. RS is one machine learning approach. Traditional statistical methods such as logistic regression can produce similar results as the RS that we use. However, because the purpose of traditional statistical methods is to interpret parameters, using RS is a much simpler method and saves time. Applying various methods of machine learning, including RS, to big data, such as claim data, will provide researchers with new insights. However, various attempts are needed.

### Interpretation

Benign breast disease is a risk factor for breast cancer and requires active screening^14^. Recently, Farland *et al*. reported that endometriosis is associated with benign breast disease, which is similar to our results^15^. There are many hypotheses about the pathogenesis of endometriosis, but there is no question that endometriosis is an estrogen-dependent disease^1^. Women with benign breast disease have a higher estrogen level than normal women, and tamoxifen, which acts against estrogen, reduces the incidence of benign breast disease^16,17^. It is therefore tempting to speculate that estrogen may play a role in linking the two diseases. Although there have been studies that extend this concept to the relationship between endometriosis and breast cancer, the association between endometriosis and breast cancer is still inconclusive because the results of studies are inconsistent^18^.

This study showed that there was a relationship between endometriosis and simple non-toxic goitre. A previous study using HIRA-NIS reported that there was no association between endometriosis and simple non-toxic goitre^4^. This result seems to be due to differences in the definition of simple non-toxic goitre. While the previous study only used diagnostic codes for simple non-toxic goitre, the present study used both diagnostic codes and thyroid-related test codes to improve the diagnostic accuracy.

There is little explanation for the association between the two diseases. For this reason, clues were found from studies on the relationship between benign breast disease and thyroid disease^19,20^. The link between benign breast disease and thyroid disease is presumed to be due to a lack of iodine^19,21^. Iodine deficiency leads to goitre by increasing TSH secretion or response^22^. On the other hand, unlike goitre, iodine deficiency seems to induce benign breast disease by increasing estrogen activity through the following two mechanisms. First, iodine deficiency reduces the metabolism of estrone or oestradiol by decreasing cytochrome P450 1A1 (CYP1A1) & 1B1 (CYP1B1). Second, iodine deficiency promotes estrogen-induced transcription by reducing the activity of BRCA1, an inhibitor of ERα transcription^21^. Because endometriosis is an estrogen-dependent disease, the link between three diseases (endometriosis, benign breast disease, and non-toxic goitre) is presumed to be iodine^1^. However, because there have been few studies on the relationship between endometriosis and iodine, further investigation of this hypothesis is needed.

Cystitis was related to endometriosis in our study. Although a previous study examined the relationship between interstitial cystitis and endometriosis, few studies have shown a relationship between endometriosis and cystitis beyond interstitial cystitis^23^.

Although poorly understood, there is a possibility that urinary tract endometriosis (UTE) may affect the relationship between endometriosis and cystitis. UTE accounts for 0.3 to 12% of total cases of endometriosis, and vesical endometriosis accounts for 80% of UTE^24^. Because the typical symptoms of UTE are cyclic haematuria and intermittent dysuria, UTE may be a risk factor for cystitis^24^.

Interstitial cystitis was not associated with endometriosis in this study (crude OR: 1.77; 95% CI: 0.73–4.32; P = 0.21). However, previous study have reported that interstitial cystitis is associated with endometriosis^23^. Because the diagnosis of interstitial cystitis is excluded from other diseases, it is possible that interstitial cystitis may be recorded as codes of other cystitis or unspecified cystitis in this study, which is a limitation of research on diseases diagnosed by exclusion.

We did not exclude specific diseases (eg. cancer) except gynaecology-related diseases. It is only selected by the RS. It is likely to be selected by the RS because the incidence of benign diseases is much higher than cancers.

## Strengths and Limitations

There are some limitations to this study. First, the claim data in this study were annual data. Therefore, these data were useful for finding related diseases with a similar age at onset, but they were insufficient for confirming the association between diseases and different ages of onset. For example, if endometriosis is associated with uterine prolapse, the HIRA-NIS data would be limited in finding associations with both diseases. Studies on the association between diseases and different ages of onset will require years of cohort data. Second, histological classification could not be applied because there was no biopsy data of related diseases. For example, benign breast tumours include several diseases, such as fibroadenoma, lipoma, and adenoma^25^. In this study, it was not possible to identify which of the benign breast tumours were more strongly associated with endometriosis. Third, the proven effects of RS in our study were not confirmed histologically but indirectly using claim data.

In conclusion, endometriosis was associated with benign breast diseases, cystitis, and non-toxic goitre. In addition, RS based on item similarities of machine learning might be a useful method for identifying the relationship between diseases. Further studies are needed for diseases other than endometriosis.

## Supplementary information

supplement table
