# Supplementary material for: Machine Learning Approach to find the relation between Endometriosis, benign breast disease, cystitis and non-toxic goiter
Source: Sci Rep. 2019 Apr 1;9:5410. doi: 10.1038/s41598-019-41973-w (PMC6443655; doi:10.1038/s41598-019-41973-w)
Supplement: Supplementary file 1 — supplement table [file 41598_2019_41973_MOESM1_ESM.docx]

**Title: Machine Learning Approach to find the relation between Endometriosis, benign breast disease, cystitis and non-toxic goiter.**

Jung Hun Lee, M.D., Ph.D.^1^; Seon-Young Kwon, M.D.^2^; Jiho Chang^3^; Jin-Sung Yuk, M.D., Ph.D.^4*^

^1^ Department of Obstetrics and Gynecology, Ewha Womans University Seoul Hospital, School of Medicine, Ewha Womans University, Seoul, Republic of Korea

^2^ Department of Family Medicine, Yonsei Spring Clinic, Changwon-si, Republic of Korea

^3^ Coupang Korean Electronic Commerce Company, Seoul, Republic of Korea

^4^ Department of Obstetrics and Gynecology, Eulji University, College of Medicine, Nowon Eulji Medical Center, Seoul, Republic of Korea

**^*^** **Correspondence:** Jin-Sung Yuk, MD, Ph.D.; Department of Obstetrics and Gynecology, Eulji University College of Medicine, Nowon Eulji Medical Center, 327, Gongneung-ro, Nowon-gu, Seoul, Zip 01830, Republic of Korea

Tel.: 82-2-970-8715, Fax; 82-303-3446-3369

E-mail: cnnsbs@naver.com

| Supplement Table 1. Thirty categories of diseases related to endometriosis recommended by the RS model based on item similarities. | | | |
| --- | --- | --- | --- |
|  |  |  |  |
| Diagnostic Code | Related Disease | Similarity Score | Rank |
| D27 | Benign neoplasm of the ovary | 0.0833 | 1 |
| D25 | Leiomyoma of the uterus | 0.0730 | 2 |
| N83 | Noninflammatory disorders of the ovary, fallopian tube and broad ligament | 0.0600 | 3 |
| N94 | Pain and other conditions associated with female genital organs and menstrual cycle | 0.0463 | 4 |
| N93 | Other abnormal uterine and vaginal bleeding | 0.0300 | 5 |
| N73 | Other female pelvic inflammatory diseases | 0.0250 | 6 |
| D50 | Iron deficiency anaemia | 0.0223 | 7 |
| N97 | Female infertility | 0.0213 | 8 |
| N92 | Excessive, frequent and irregular menstruation | 0.0204 | 9 |
| N85 | Other noninflammatory disorders of the uterus, except the cervix | 0.0189 | 10 |
| N84 | Polyps of the female genital tract | 0.0181 | 11 |
| N76 | Other inflammation of the vagina and vulva | 0.0174 | 12 |
| N72 | Inflammatory disease of the cervix uteri | 0.0163 | 13 |
| B37 | Candidiasis | 0.0125 | 14 |
| N71 | Inflammatory disease of the uterus, except the cervix | 0.0122 | 15 |
| D24 | Benign neoplasm of the breast | 0.0122 | 16 |
| N60 | Benign mammary dysplasia | 0.0119 | 17 |
| N64 | Other disorders of the breast | 0.0113 | 18 |
| D64 | Other anaemias | 0.0107 | 19 |
| N30 | Cystitis | 0.0106 | 20 |
| R10 | Abdominal and pelvic pain | 0.0102 | 21 |
| N70 | Salpingitis and oophoritis | 0.0102 | 22 |
| N87 | Dysplasia of the cervix uteri | 0.0097 | 23 |
| D39 | Neoplasm of uncertain or unknown behaviour of female genital organs | 0.0086 | 24 |
| N91 | Absent, scanty and rare menstruation | 0.0085 | 25 |
| Z98 | Other postsurgical states | 0.0082 | 26 |
| T81 | Complications of procedures, NEC | 0.0076 | 27 |
| N95 | Menopausal and other perimenopausal disorders | 0.0075 | 28 |
| D26 | Other benign neoplasms of the uterus | 0.0074 | 29 |
| E04 | Other non-toxic goitre | 0.0073 | 30 |
|  |  |  |  |
| RS: recommender system | | | |

| Supplement Table 2. Endometriosis-related candidate diseases selected in this study | |
| --- | --- |
|  |  |
| Diagnostic Code | Related Disease |
| **D24** | **Benign neoplasm of the breast** |
| **N60** | **Benign mammary dysplasia** |
| N600 | Solitary cyst of the breast |
| N601 | Diffuse cystic mastopathy |
| N602 | Fibroadenosis of the breast |
| N603 | Fibrosclerosis of the breast |
| N604 | Mammary duct ectasia |
| N608 | Other benign mammary dysplasias |
| N609 | Benign mammary dysplasia, unspecified |
| **N64** | **Other disorders of breast** |
| N640 | Fissure and fistula of the nipple |
| N641 | Fat necrosis of the breast |
| N642 | Atrophy of the breast |
| N643 | Galactorrhoea not associated with childbirth |
| N644 | Mastodynia |
| N645 | Other signs and symptoms in the breast |
| N648 | Other specified disorders of the breast |
| N649 | Disorder of the breast, unspecified |
| **N30** | **Cystitis** |
| N300 | Acute cystitis |
| N301 | Interstitial cystitis |
| N302 | Other chronic cystitis |
| N303 | Trigonitis |
| N304 | Irradiation cystitis |
| N308 | Other cystitis |
| N309 | Cystitis, unspecified |
| **E04** | **Other non-toxic goitre** |
| E040 | Non-toxic diffuse goitre |
| E041 | Non-toxic single thyroid nodule |
| E042 | Non-toxic multinodular goitre |
| E048 | Other specified non-toxic goitre |
| E049 | Non-toxic goitre, unspecified |
| **D50** | **Iron deficiency anaemia** |
| D500 | Iron deficiency anaemia secondary to blood loss (chronic) |
| D501 | Sideropenic dysphagia |
| D508 | Other iron deficiency anaemias |
| D509 | Iron deficiency anaemia, unspecified |
| **D64** | **Other anaemias** |
| D640 | Hereditary sideroblastic anaemia |
| D641 | Secondary sideroblastic anaemia due to disease |
| D642 | Secondary sideroblastic anaemia due to drugs and toxins |
| D643 | Other sideroblastic anaemias |
| D644 | Congenital dyserythropoietic anaemia |
| D648 | Other specified anaemias |
| D649 | Anaemia, unspecified |

| Supplement Table 3. Adjusted logistic regression analysis of endometriosis-related candidate diseases using middle-class diagnostic codes according to age per 5-years | | | | | | |
| --- | --- | --- | --- | --- | --- | --- |
|  |  |  |  |  |  |  |
|  | 15~24 years ^a^ | | 25~34 years ^a^ | | 35~45years ^a^ | |
|  | OR (95% CI) | P-value | OR (95% CI) | P-value | OR (95% CI) | P-value |
| Age per 5 years | 7.01 (5.00-9.82) | <0.01 | 1.09 (0.99-1.20) | 0.08 | 0.99 (0.91-1.08) | 0.85 |
| Data year | 1.02 (0.97-1.06) | 0.52 | 1.00 (0.98-1.03) | 0.84 | 1.01 (0.99-1.04) | 0.19 |
| Low SES | 0.53 (0.33-0.86) | <0.01 | 1.02 (0.51-2.04) | 0.96 | 0.65 (0.49-0.88) | <0.01 |
| Benign neoplasm of the breast | 0 (0-0) | <0.01 | 4.25 (2.30-7.85) | <0.01 | 2.14 (1.59-2.88) | <0.01 |
| Benign mammary dysplasia | 0 (0-0) | <0.01 | 1.55 (1.00-2.41) | 0.05 | 2.16 (1.35-3.44) | <0.01 |
| Other disorders of the breast | 0 (0-0) | <0.01 | 1.45 (1.09-1.92) | 0.01 | 1.87 (1.30-2.71) | <0.01 |
| Cystitis | 1.28 (0.76-2.14) | 0.35 | 1.50 (1.23-1.84) | <0.01 | 1.45 (1.23-1.71) | <0.01 |
| Other non-toxic goitre | 0.79 (0.20-3.18) | 0.74 | 1.25 (0.91-1.72) | 0.17 | 1.73 (1.16-2.58) | <0.01 |
| Iron deficiency anaemia | 2.53 (1.60-3.99) | <0.01 | 3.42 (2.25-5.20) | <0.01 | 3.09 (2.49-3.84) | <0.01 |
| Other anaemias | 0.91 (0.34-2.46) | 0.86 | 1.61 (0.76-3.41) | 0.21 | 2.59 (1.79-3.74) | <0.01 |
|  |  |  | | | | |
| CI, confidence interval; OR, odds ratio; SES, socioeconomic status | | | | | | |
|  | | | | | | |

^a^ Analysis was adjusted for all variables in the table (endometriosis ~ age per 5 years + data year + low SES + benign neoplasm of breast + benign mammary dysplasia + other disorders of the breast + cystitis + other non-toxic goitre + iron deficiency anaemia + other anaemias).

| Supplement Table 4. Logistic regression for each disease adjusted for age per 5 years and data year | | | | | | | |
| --- | --- | --- | --- | --- | --- | --- | --- |
|  |  | |  | |  |  |  |
| Diseases | | Adjusted for age per 5 years, data year, and SES ^a^ | | | |  |  |
|  | | OR (95% CI) | | P-value | |  |  |
| Benign neoplasm of the breast | | 2.72 (1.88-3.94) | | <0.01 | |  |  |
| Diffuse cystic mastopathy | | 2.34 (1.08-5.09) | | 0.03 | |  |  |
| Fibroadenosis of the breast | | 0.95 (0.47-1.92) | | 0.89 | |  |  |
| Other benign mammary dysplasias | | 1.26 (0.69-2.30) | | 0.44 | |  |  |
| Unspecified benign mammary dysplasia | | 2.83 (1.44-5.57) | | <0.01 | |  |  |
| Mastodynia | | 1.38 (0.91-2.09) | | 0.12 | |  |  |
| Other signs and symptoms in the breast | | 2.97 (1.65-5.35) | | <0.01 | |  |  |
| Other specified disorders of the breast | | 2.05 (1.12-3.76) | | 0.02 | |  |  |
| Unspecified disorder of the breast | | 2.21 (1.54-3.15) | | <0.01 | |  |  |
| Acute cystitis | | 1.41 (1.22-1.63) | | <0.01 | |  |  |
| Other chronic cystitis | | 1.21 (0.76-1.94) | | 0.42 | |  |  |
| Other cystitis | | 2.92 (1.74-4.90) | | <0.01 | |  |  |
| Unspecified cystitis | | 1.85 (1.42-2.42) | | <0.01 | |  |  |
| Non-toxic diffuse goitre | | 1.06 (0.50-2.24) | | 0.88 | |  |  |
| Non-toxic single thyroid nodule | | 1.73 (1.21-2.46) | | <0.01 | |  |  |
| Non-toxic multinodular goitre | | 1.73 (1.09-2.73) | | 0.02 | |  |  |
| Unspecified non-toxic goitre | | 1.54 (0.65-3.65) | | 0.33 | |  |  |
| Iron deficiency anaemia secondary to blood loss (chronic) | | 6.25 (3.52-11.08) | | <0.01 | |  |  |
| Other iron deficiency anaemias | | 2.75 (1.82-4.15) | | <0.01 | |  |  |
| Iron deficiency anaemia, unspecified | | 3.09 (2.46-3.87) | | <0.01 | |  |  |
| Other specified anaemias | | 3.03 (1.80-5.11) | | <0.01 | |  |  |
| Anaemia, unspecified | | 2.43 (1.70-3.47) | | <0.01 | |  |  |
|  |  | |  | |  |  |  |
| SES, socioeconomic status  Diseases with a prevalence of less than 0.1% in both groups are not shown in the table. | | | | | | |  |

^a^ Analysis was adjusted for age per 5 years, data year, and each disease (endometriosis ~ age per 5 years + data year + low SES + one disease).
